# Validation of the biomarker toolkit using diagnostic colorectal cancer biomarkers – an evidence-based tool to support clinical adoption of biomarkers

**DOI:** 10.1186/s12967-026-08143-9

**Published:** 2026-05-19

**Authors:** Alice E. Baggaley, Yue Wu, Katerina-Vanessa Savva, Komal Khan, Prerana Gogoi, Hateem Rafeeque, Maxime Giot, Marina Siakalli, Constantinos Panayiotou, Melody Zhifang Ni, Christopher J. Peters

**Affiliations:** 1 Department of Surgery and Cancer, Imperial College London, London, UK; 2 Clinical Research Fellow Academic, Department of Surgery and Cancer, Hammersmith Hospital, 72 Du Cane Road, London, W12 0HS UK

**Keywords:** Biomarkers, Translational research, Clinical utility, Colorectal cancer

## Abstract

**Background:**

Biomarkers offer valuable insights into diseases and bodily functions, yet many fail to achieve clinical implementation. Our recent research revealed a substantial translational gap, with only 0.94% of prognostic breast cancer biomarkers and 0.14% of diagnostic colorectal cancer biomarkers reaching clinical practice. To address this challenge, we developed the Biomarker Toolkit, designed to enhance translational outcomes by identifying cancer biomarkers most likely to succeed and highlighting areas requiring further research. This study aims to validate the Toolkit using a new dataset of diagnostic colorectal cancer biomarkers.

**Methods:**

Systematic literature searches were conducted to identify clinical studies for successful and stalled diagnostic colorectal cancer biomarker groups. ‘Successful’ biomarkers were defined as those approved for diagnostic use in colorectal cancer by regulatory bodies. For the stalled cohort, a random selection of biomarkers associated with > 5 publications was drawn from a previous review, until the number of clinical studies in each cohort was equal. Each clinical study was examined for the presence or absence of 125 attributes present in the Toolkit, with a binary score (1 or 0) assigned accordingly. Total and category scores were calculated and compared between the successful and stalled biomarker groups, using Mann Whitney U Test, binary logistic and Cox regression analyses.

**Results:**

Using the Toolkit, successful diagnostic colorectal cancer biomarkers demonstrated significantly higher total scores compared to the stalled group (44.7% versus 25.1%, *p* < 0.001). Successful biomarkers exhibited higher clinical utility scores even before receiving regulatory approval (42.1% versus 20.2%, *p* < 0.001). Additionally, the frequency and diversity of clinical utility publications for successful biomarkers continued to rise beyond the initial five-year period following the first biomarker publication.

**Conclusions:**

This study provides independent validation of the Biomarker Toolkit, having been initially validated using prognostic breast cancer biomarkers. The significantly higher Toolkit scores observed in successful diagnostic colorectal cancer biomarkers, particularly in clinical utility, even before regulatory approval, underscore the Toolkit’s strong potential for predicting translational success.

**Supplementary Information:**

The online version contains supplementary material available at 10.1186/s12967-026-08143-9.

## Background

Biomarkers are measurable indicators within the body that can signal normal or abnormal processes. They are used across a wide spectrum of diseases for various purposes. The Food and Drug Administration (FDA) Biomarker Working Group has produced the BEST resource (Biomarkers, EndpointS, and other Tools) to define different types of biomarkers: susceptibility, diagnostic, monitoring, prognostic, predictive, pharmacodynamic/response, and safety [1]. Biomarker development generally progresses through several phases, from discovery through to adoption, including assay validation, clinical validation and clinical utility. However, this pipeline is complex, and multiple factors contribute to stalled advancement, including poorly constructed studies, institutional silos and unclear regulatory requirements [2, 3].

Recent evidence from our group has quantified the extent of stalled progress in biomarker development. When investigating all prognostic breast cancer biomarkers in the literature, only 0.94% were found to have been successfully translated into clinical use [4]. A similar study focused on diagnostic colorectal cancer (CRC) biomarkers, identified a total of 2910 biomarkers in the literature. Of these, only four had been approved for use, reflecting a translation rate of just 0.14% [5].

This significant translational gap not only reflects considerable waste but also highlights the potential for successful implementation of additional CRC diagnostic biomarkers that may have stalled but still have promise. Since no single biomarker is likely to achieve perfect sensitivity and specificity, a diverse range of successfully adopted CRC diagnostic biomarkers may enhance clinical decision-making. This is particularly important given the current CRC diagnostic pathway’s capacity constraints for colonoscopy [6].

The Imperial Biomarker Group has developed the Biomarker Toolkit, using systematic literature searches, semi-structured interviews and Delphi survey methodologies [7], to increase the translational success from biomarker discovery to clinical use. In brief, the Toolkit is a checklist comprising 125 attributes which are associated with successful biomarkers, categorised into three main categories: analytical validity (AV), clinical validity (CV) and clinical utility (CU). It assesses reporting of primary clinical studies that directly evaluate the performance of biomarkers in their intended clinical use. The outputs from the Toolkit, in the form of a total and category score, help identify biomarkers most likely to be successfully translated and highlight areas needing further research and development. The Toolkit is designed to evaluate all types of biomarkers, as it focuses on the methodologies employed to facilitate clinical translation, rather than specific sampling technologies utilised in biomarker development.

The Biomarker Toolkit has previously demonstrated that successfully implemented prognostic biomarkers for breast and colorectal cancer, even before receiving FDA approval, consistently report higher Toolkit scores compared to their stalled counterparts [7].

This novel study aims to validate the Biomarker Toolkit with a different biomarker type (diagnostic versus prognostic), focusing on diagnostic CRC biomarkers, using an independent dataset. We hypothesise that biomarkers successfully translated into clinical practice will score more highly with the Toolkit. We aim to compare successfully translated diagnostic CRC biomarkers with a random selection of stalled biomarkers, as well as assess interrater reliability of the Toolkit.

## Methods

### Selection of primary clinical studies

Systematic literature searches were conducted to identify relevant diagnostic CRC biomarkers for the application of the Toolkit. Biomarkers were defined as ‘successful’ if they had received approval from the FDA or National Institute for Health and Care Excellence (NICE), for use in CRC diagnosis. Four successful diagnostic CRC biomarkers were identified. Stalled biomarkers were selected from a database of 2910 published diagnostic CRC biomarkers compiled through a systematic review in previously published group work [5]. A sample of stalled biomarkers, each with over five publications identified in our database, were chosen using random number generation in Excel. Stalled diagnostic CRC biomarkers associated with fewer than 5 publications were excluded, as we wished to ensure that a systematic search would yield a sufficient volume and variety of studies over time to make a comparison across the stalled and successful cohort as robust as possible. This process was repeated until the number of clinical studies in both groups was comparable. In total, eleven independent systematic literature searches were conducted in Medline and Embase, covering the period from 1946 to 2023. Relevant keywords were used to identify studies for each diagnostic CRC biomarker (Additional file 1: Table S1, Table S2).

The volume of clinical studies retrieved for one of the successful diagnostic CRC biomarkers, Faecal Immunochemical Test (FIT) (*n* = 509) (Additional file 1: Table S1), greatly outnumbered the next highest frequency successful biomarker (gFOBT, *n* = 123). Given time constraints of the project, it was not feasible to score the entire FIT dataset, and therefore a sample of 20% of these studies, which spanned the entire publication timeline of FIT, were selected. This brought the number of clinical studies to be scored in line with gFOBT. In order to reduce bias and provide comprehensive representation of the FIT publication history, the selection process involved sorting all published studies in chronological order, then a random sequence of numbers spanning the publication period was generated and used to select the studies for scoring. This approach aimed to provide a comprehensive representation and enable the exploration of how reporting on the biomarker evolved over time.

### Abstract and full paper screening

All primary clinical studies evaluating the relevant biomarker in isolation (either in vitro or in vivo) for CRC diagnosis were included. These included studies investigating both CRC and its associated premalignant lesions (e.g., advanced adenomas) as a combined outcome. For successful CRC diagnostic biomarkers, only the NICE and FDA-approved tissue formats were included. Conversely, for stalled CRC diagnostic biomarkers, any tissue format was permissible, to maximise the number of clinical studies available as evidence.

Additional exclusion criteria applied during screening included: conference abstracts; studies not published in English; paper types other than primary studies; and studies where the relevant biomarker was not directly evaluated for CRC diagnosis. Duplicate citations were removed during the systematic search. All abstracts were screened on the Covidence platform by two reviewers (YW, AEB, KK, PG, MG, JK) [8]. Conflicts during the screening process were resolved with an independent reviewer (KVS).

Included studies were classified into three types: clinical, assay validation and clinical utility. Within the clinical utility category, studies were further divided into five sub-types (Table 1). If a study significantly addressed multiple sub-types, it was categorised under each relevant type separately.

**Table 1:** Description of paper sub-types

Study Type	Definition	Inclusion	Exclusion	Example paper
Clinical	Assesses performance of selected biomarker for CRC diagnosis.	Link made between biomarker and diagnosis of CRC, *in vivo/in vitro* samples	Biomarker candidate only assessed as a panel. Reviews, case studies, conference abstracts, letters, commentaries, not published in English	PMID: 34933958
Assay Validation	Assesses the technical aspects of assay technology used for selected biomarker.	Accuracy of assay performance detecting biomarker candidate, i.e., reproducibility of the assay	Outcome being assessed is solely CRC diagnosis	PMID: 20804913
Clinical Utility				
- Cost-effectiveness	Assesses additional cost benefits and incremental clinical utility achieved by use of selected biomarker compared to costs of comparable current clinical tools for CRC diagnosis.	Study measures biomarker being used as diagnostic CRC tool, using CE outcomes	Reviews, case studies, conference abstracts, letters, commentaries, not published in English	PMID: 28733262
- Human factor	Assesses participants’ opinions regarding use of selected biomarker. This includes exploring participants’ preference for using biomarker compared to other methods of CRC diagnosis and usability of biomarker for clinical testing.	Explores patient/clinician acceptability of test, and/or any potential hazards	Reviews, case studies, conference abstracts, letters, commentaries, not published in English	PMID: 24636031
- Decisional analysis	Assesses applicability of selected biomarker to patient pathway, including its potential to influence clinicians’ diagnostic approaches.	Uses formal decision models such as decision trees	Reviews, case studies, conference abstracts, letters, commentaries, not published in English	PMID: 22307927
- Feasibility/ implementation	Assesses utilisation of selected biomarker in real world conditions.	Studies examining the impact of demographic and epidemiological factors, participation rates and barriers to adoption	Reviews, case studies, conference abstracts, letters, commentaries, not published in English	PMID: 11879329
- Utility	Encompasses any other clinical utility studies not covered by the above sub-types.		Reviews, case studies, conference abstracts, letters, commentaries, not published in English	PMID: 18462248

### Application of the Biomarker Toolkit to diagnostic CRC biomarkers

The Biomarker Toolkit was applied to all primary clinical studies evaluating both successful and stalled CRC diagnostic biomarkers [7]. The Toolkit consists of 125 attributes associated with successful biomarker translation, grouped into three main categories: Analytical Validity (AV), Clinical Validity (CV), and Clinical Utility (CU). For each biomarker, a score of ‘1’ was assigned if an attribute was reported, and ‘0’ if absent. Methodological details from referenced studies were consulted to inform the scoring, particularly for analytical techniques and patient cohort selection.

**Fig. 1:**
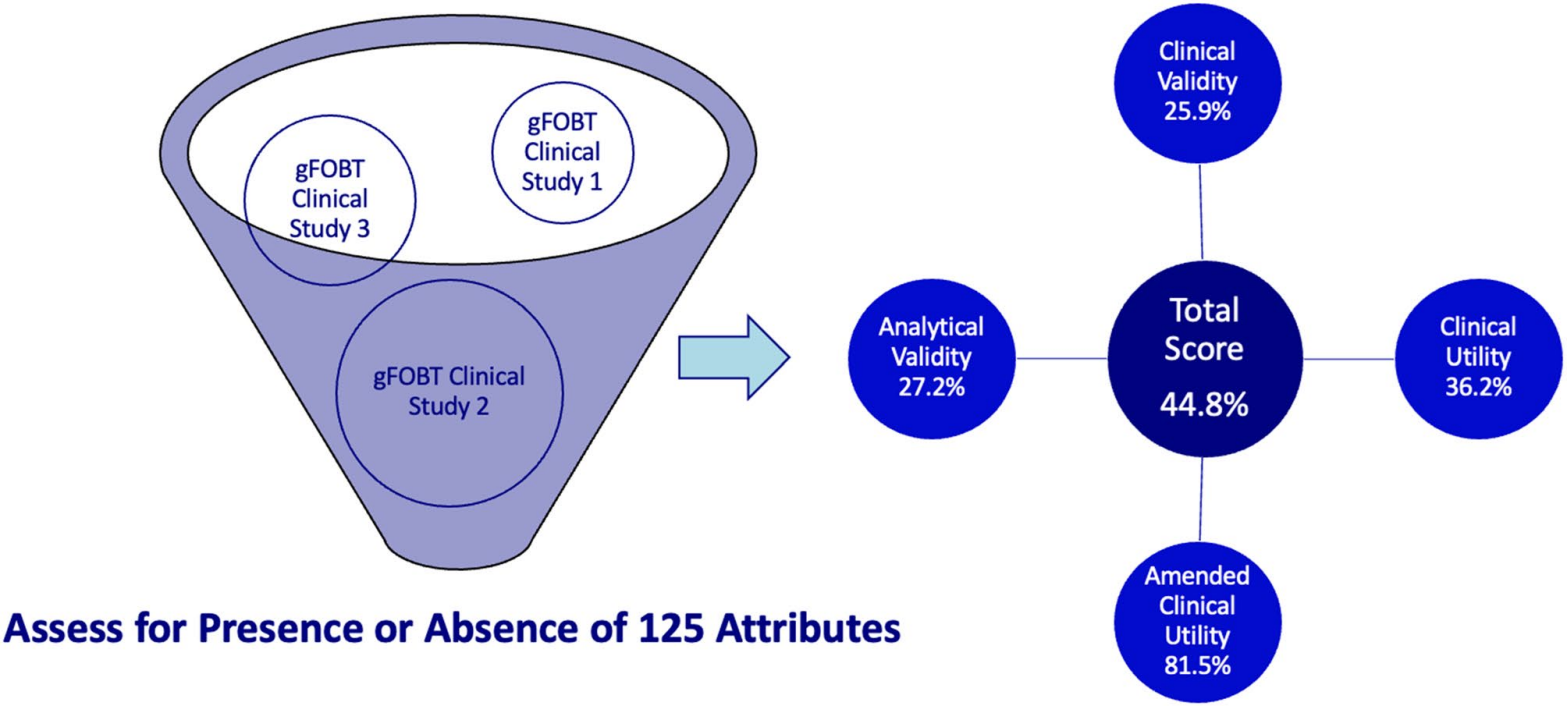
Overview of the process for generating toolkit scores from primary clinical studies

The calculation of Toolkit scores has been described at length in the original publication on the development of the Biomarker Toolkit [7]. In short, scores for each biomarker were calculated using a four-step process. First, an average score was obtained for all sub-categories (*n* = 44). This average contributed to the score for each of the three main categories (AV, CV, CU). Next, an amended CU score was generated by accounting for additional clinical utility study sub-types alongside clinical studies (Table 1). Finally, an overall average score was derived from the average score of each category (Fig. 1). The amended CU score considers the presence of any clinical utility studies published prior to the clinical study being evaluated, with a binary score. The clinical utility studies are not themselves assessed with the Biomarker Toolkit.

### Quality check of the Biomarker Toolkit

A random subset (targeting 20%) of clinical studies underwent double scoring by second reviewers (YW, AEB) to evaluate interrater reliability and internal validity. If discrepancies between first and second reviewers were less than 12%, consistent with previous findings of discrepancies from double scoring [7], the scores from the first reviewer were used for quantitative analysis within the Toolkit. Cohen’s kappa coefficient was also calculated to quantify the consistency of assessments between reviewers.

### Statistical analysis

The D’Agostino normality test was used to assess the normality of the data. The Mann-Whitney U test was then employed to compare differences between successful and stalled CRC biomarkers in clinical validity, analytical validity and amended clinical utility categories. Additionally, the scores of successful biomarkers before and after regulatory approval were compared to evaluate the impact of regulatory approval on their performance.

Binary logistic regression was performed to assess the association between biomarker implementation status (a binary outcome) and scores from (i) the total score, (ii) main categories (analytical validity, clinical validity, amended clinical utility), and (iii) sub-categories. Cox regression analysis was used to account for the effect of time, measuring the interval between study publication date and first publication of the relevant biomarker, alongside scores, with successful regulatory approval of a biomarker for diagnostic use in colorectal cancer defined as the ‘event’ for both models. Sub-category level analyses were prespecified as exploratory given the limited number of approved biomarkers; with effect estimates interpreted primarily in terms of directionality rather than as definitive independent predictors. To minimise post-event bias, successful biomarkers with clinical studies published after FDA approval were excluded, so only scores from pre-approval contributed to regression analyses. Collinearity and proportional hazards assumptions were assessed prior to model interpretation.

A sensitivity analysis was performed to assess the influence of the sampling of FIT clinical studies and ensure robustness of our results. Mann Whitney U tests were used to comparing total and sub-category scores of the stalled cohort vs. the successful cohort with the FIT scores removed. An auxiliary analysis was performed to check robustness of results with regards to clinical study publication frequency. Successful and stalled biomarkers were separated into three strata of low publication count (*n* < 30), medium publication count (30 ≤ *n* ≥ 70), and high publication count (*n* > 70). One successful and stalled biomarker from each strata was randomly selected to create a sample (*n* = 6 biomarkers), where total Toolkit scores across each cohort were compared. This analysis was performed in replicate, with ten random samples.

Calculated *p-value <* 0.05 was considered to denote significant differences. R studio (Version 2022.12.0 + 353) was used to complete all statistical analyses [9]. Descriptive analyses were undertaken to assess differences in the quantity and type of publications related to successful CRC diagnostic biomarkers before and after FDA approval.

## Results

Diagnostic CRC biomarker literature searches identified 291 clinical studies for successful biomarkers (Guaiac Fecal Occult Blood Test (gFOBT) [10], *n* = 123; Fecal Immunochemical Test (FIT) [11], *n* = 101; methylated Septin9 (mSEPT9) [12], *n* = 41; Cologuard [13], *n* = 26), and 230 clinical studies for stalled biomarkers (ALU Based Cell Free DNA (ALU), *n* = 18; Fusibacterium Nucleatum (Fn), *n* = 43; M2 Pyruvate Kinase (M2PK), *n* = 38; MicroRNA-200c (Mir200c), *n* = 17; MicroRNA-21 (Mir21), *n* = 75; Transferrin, *n* = 11). A PRISMA table detailing all study types retrieved for each biomarker is provided in Additional file 1: Table S1.

**Fig. 2:**
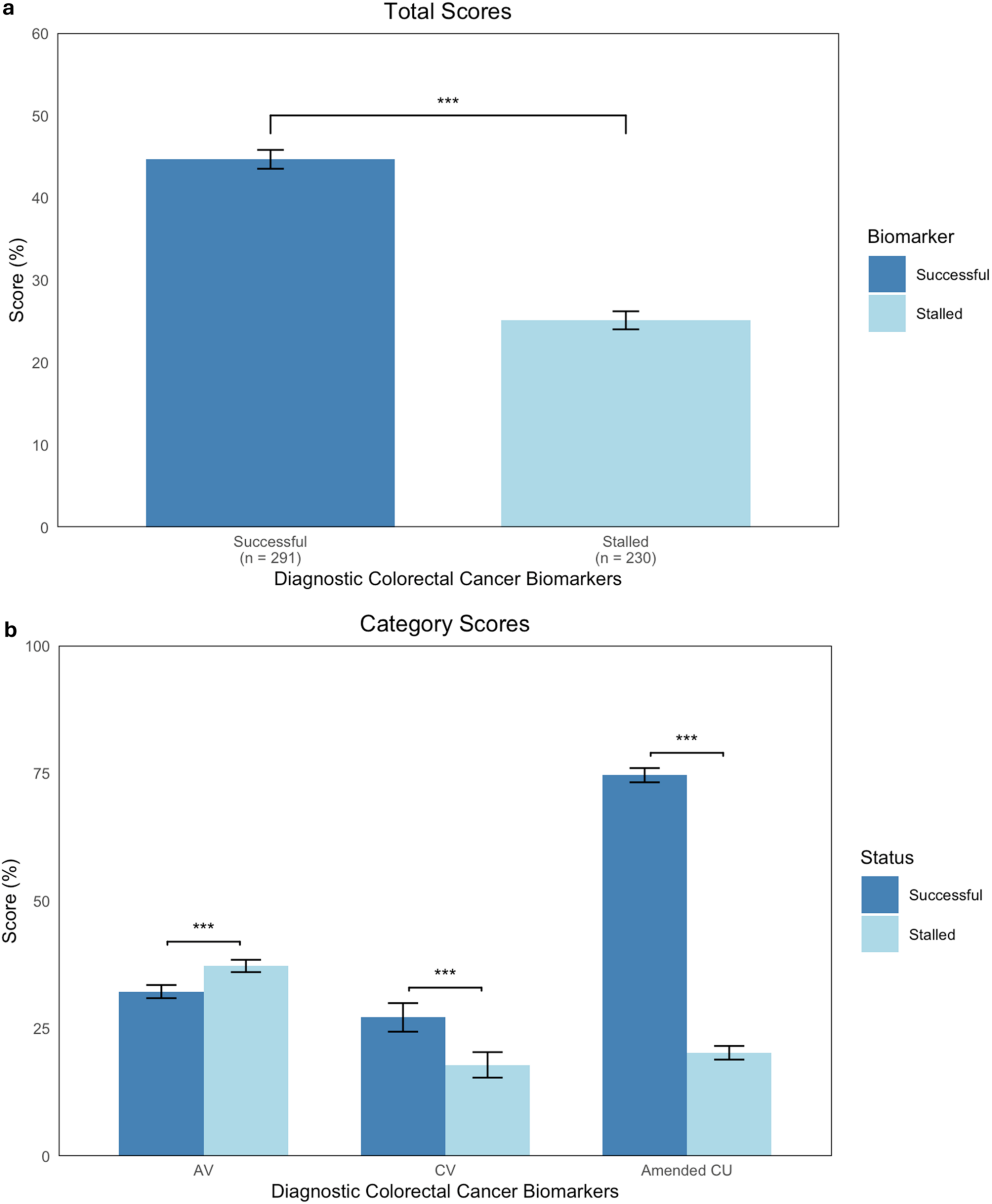
Scores of Successful and Stalled Diagnostic CRC Biomarkers. **a** Average total scores between successful (44.7% / 95% CI [43.5% − 45.8%]) vs. stalled biomarkers (25.1% / 95% CI [24.0% − 26.2%]); Mann-Whitney U test: *p* < 0.001. **b** Average main category scores for AV (successful: 32.2% / 95% CI [30.9% − 33.5%] vs. stalled: 37.2% / 95% CI [36.0% − 38.5%]; Mann-Whitney U test: *p* < 0.001); CV (successful: 27.2% / 95% CI [24.4% − 30.0%] vs. stalled: 17.9% / 95% CI [15.4% − 20.4%]; Mann-Whitney U test: *p* < 0.001) and Amended CU (successful 74.6% / 95% CI [73.2% − 76.0%] vs. Stalled: 20.2% / 95% CI [18.9% − 21.6%]; Mann-Whitney U test: *p* < 0.001). Asterisks denote level of significance where: *** *p* < 0.001. Error bars denote CI AV, Analytical Validity; CV, Clinical Validity; Amended CU, Amended Clinical Utility

The average total scores of successful diagnostic CRC biomarkers were significantly higher in comparison to the stalled biomarker group (Fig. 2a / 44.7% versus 25.1% / Mann-Whitney U test: *p* < 0.001). Scores across all main categories were significantly different between successful and stalled biomarkers when compared as a group (Fig. 2b) and individually (Additional file 1: Figure S5). The amended clinical utility score had the biggest score difference (Fig. 2b / 74.6% versus 20.2% / Mann-Whitney U test: *p* < 0.001) (Additional file 1: Table S4).

Binary logistic and Cox proportional hazards regression analyses demonstrated that both clinical validity (OR = 1.089, 95% CI 1.012–1.172; HR = 1.066, 95% CI 1.008–1.126) and amended clinical utility (OR = 1.077, 95% CI 1.012–1.147; HR = 1.032, 95% CI 1.007–1.058) were significantly associated with an increased likelihood of approval (Additional file 1: Table S3). No violations of collinearity or proportional hazards assumptions were detected.

**Fig. 3:**
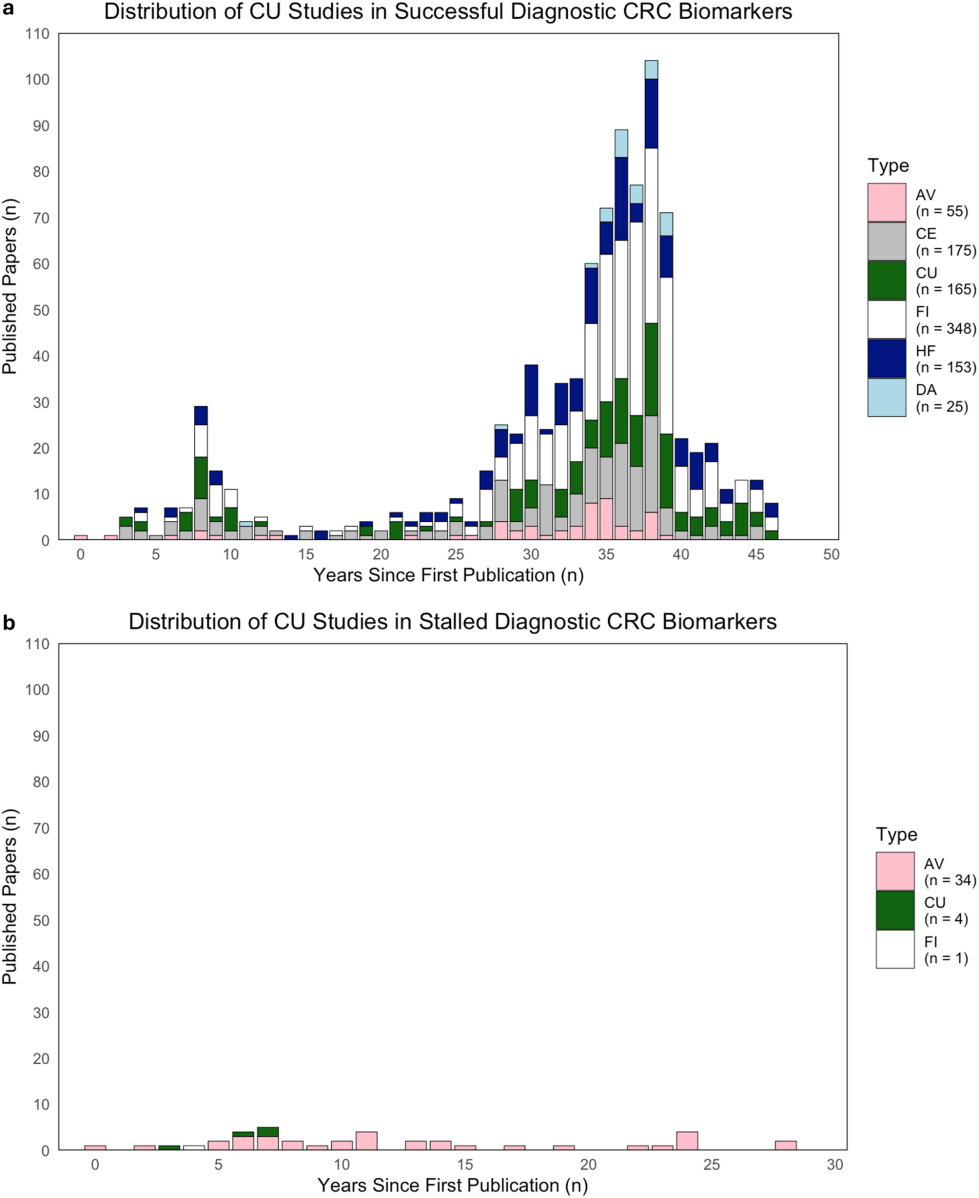
Publication frequency of clinical utility studies since first publication of each biomarker. **a** Aggregate publication frequency of successful diagnostic CRC biomarkers. **b** Aggregate publication frequency of stalled diagnostic CRC Biomarkers. AV, Analytical Validity; CE, Cost-effectiveness; CU, Clinical Utility; FI, Feasibility and Implementation; HF, Human Factors; DA, Decisional Analysis

**Fig. 4:**
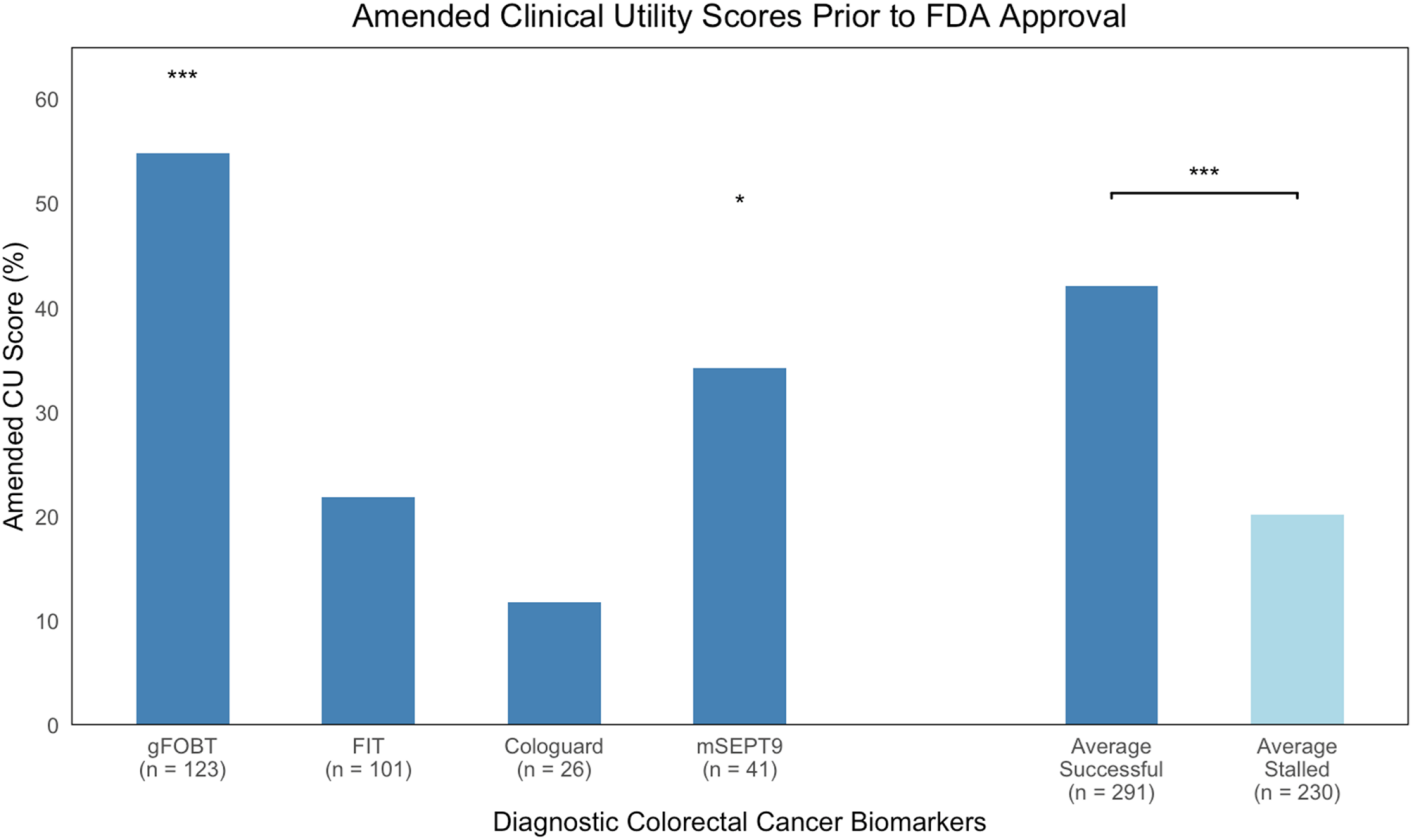
Amended clinical utility scores of successful diagnostic colorectal cancer biomarkers prior to FDA approval. Mann Whitney U test was performed to compare the clinical utility scores of successful diagnostic colorectal cancer biomarkers prior to regulatory approval with the average scores of stalled biomarkers. Asterisks denotes level of significance where: * *p* < 0.05, *** *p* < 0.001

With regards to publication frequency, it was noted that the annual publication rate of clinical utility papers was much higher in successful biomarkers (Fig. 3). This rate significantly increased for Cologuard, FIT and gFOBT biomarkers after receiving FDA approval as a diagnostic CRC biomarker (Additional file 1: Fig. S6). Average amended clinical utility scores in successful biomarkers, calculated prior to the year of respective FDA approval, were still significantly greater than amended clinical utility scores of stalled biomarkers (Fig. 4 / 42.1% versus 20.2% / Mann-Whitney U test: *p* < 0.001).

## Discussion

This study provides independent validation of the Biomarker Toolkit using a dataset of diagnostic colorectal cancer biomarkers. Application of the Toolkit to these biomarkers demonstrates significantly higher total scores in the four successfully implemented biomarkers compared to the random selection of stalled biomarkers, even prior to FDA or NICE approval. Of note, the application of the Toolkit was conducted prior to the recent approval of a fifth diagnostic CRC biomarker, the Shield test [14], which is therefore not included in this analysis.

As outlined in the introduction, the Biomarker Toolkit was initially developed and validated by our group using two sets of prognostic biomarkers: breast and colorectal cancer [7]. For the Toolkit to gain adoption by relevant stakeholders – such as academics, industry, funders, and commissioners – it is crucial that it undergoes robust validation for other biomarker uses, to ensure high confidence in its application. Internal validation of the Toolkit included independent double scoring of over 15% of the reviewed papers, demonstrating strong interrater reliability, with less than 8% difference between the scores of reviewer 1 and reviewer 2. Overall agreement between the two raters was substantial, with a Cohen’s Kappa coefficient (κ) of 0.823. These findings are consistent with results from the original validation, which showed less than 12% difference between the two sets of scores [7].

As illustrated in Fig. 3 and Additional file 1: Fig. S6, successful biomarkers have a significantly higher number and variety of clinical utility studies published compared to stalled ones. This trend emerged early in the publication timeline, with regulatory approval occurring, on average, 10 years after the first publication. The difference in amended clinical utility scores is observed before FDA approval (42.1% versus 20.2% / Mann-Whitney U test: *p* < 0.001), and then further increases afterwards (74.6% versus 20.2% / Mann-Whitney U test: *p* < 0.001). Since calculation of the amended Clinical Utility score accounts for the presence of published clinical utility studies, previous criticism suggested that approved biomarkers may score higher in this domain simply due to the increased number of associated CU papers, however this is not the case (Additional file 1: Fig. S6). It is important to emphasise that assessment of a cancer biomarker’s clinical utility, and related sub-type studies, should ideally be conducted in parallel with biomarker development, as they often constitute part of the regulatory evidence required for approval.

Interestingly, analytical validity (AV) scores were higher in the stalled biomarker group compared to the successful group (Fig. 2b). However, further analyses using both binary logistic and Cox-regression models revealed that AV score does not significantly affect the likelihood of a diagnostic CRC biomarker’s success (Additional file 1: Table S3). A possible hypothesis is that efforts to refine a biomarker’s performance focuses on repeating similar clinical studies, thereby increasing AV scores without necessarily advancing the biomarker through the next developmental stages.

While weighting of attributes and sub-categories were considered during development of the Toolkit, it did not significantly enhance differentiation between stalled and successful biomarkers and added unnecessary complexity [7]. Our findings here support that weighting is not essential to distinguish biomarker success. As we continue validating the Toolkit across other biomarker types, non-cancer settings, and real-world implementation studies, we may revisit this approach to account for how weighting can vary based on stakeholder needs and the stage of biomarker development.

There were some limitations of this study regarding biomarker selection and sampling. For the cohort of stalled biomarkers, only those featured in at least five publications in our previous review were included. This criterion ensured that a sufficient number and variety of studies could be captured, allowing for a meaningful comparison of successful and stalled diagnostic CRC biomarkers using the Toolkit. We acknowledge this process was likely to introduce bias, although in the favour of the stalled cohort, as biomarkers with few papers are unlikely to be able to score across the breadth of the Biomarker Toolkit. Biomarkers with only one paper are also more likely to have been abandoned by researchers or to be at a very early developmental stage. By selecting stalled biomarkers that have been further developed, we hoped to more robustly validate the Biomarker Toolkit as a useful and practical tool for stakeholders. Stratified random sampling was undertaken to check robustness of results with regards to publication frequency. Results were consistent and robust across all replicates (*n* = 10) (Total score, successful vs. stalled, Mann-Whitney U: *p* < 0.0001).

One of the successful biomarkers (FIT) had a sample of their clinical studies scored; a random selection distributed across the chronological timeline was used to ensure representation of the full dataset. However, due to citation chaining during the data collection, the final FIT sample was enriched with early landmark publications. This could have introduced bias to the successful biomarker cohort scores. To test the robustness of our main findings, we performed a sensitivity analysis removing the FIT scores from the successful cohort (total scores for successful biomarkers without FIT vs. stalled score (44.3% vs. 25.1% / Mann Whitney U test: *p* < 0.001)). A critique of the validation of the Biomarker Toolkit so far, is that is has been retrospective in nature. Ideally, we would prospectively score a large number of biomarkers, at multiple time points along their development, and track which ones were successfully implemented in clinical practice. However, given most biomarkers take a minimum of 10 years from discovery to translation, this approach is not currently feasible for our innovation. Instead, we have collaborated with Cancer Research Horizons to apply the Toolkit to a number of early biomarkers CRUK have funded, and provide a translational readiness report. We hope to build a bank of these real-world use cases and can continue to apply the Toolkit as the biomarkers develop, creating a prospective validation dataset.

Looking ahead, future efforts will also include the addition of a fourth category to the Toolkit: clinical and biological rationale. Multi-modal methodology will be applied to gather expert consensus regarding desirable clinical and biological rationale characteristics for successful biomarkers. The clinical rationale will cover aspects such as whether a biomarker matches an unmet need, or where it fits within the clinical pathway. The biological rationale covers whether a putative biomarker makes scientific sense, or whether it will be biologically plausible. These characteristics will be collated into the broader Toolkit checklist to provide crucial additional information for stakeholders, with potential impact right at the beginning of the developmental pipeline. We also aim to use machine learning to establish predefined thresholds for each stage of biomarker development and provide tailored, actionable guidance to facilitate progression at each step of the biomarker developmental pipeline.

The Toolkit will soon be available as a tool through the Imperial College NIHR HealthTech Research Centre (HRC) In Vitro diagnostics website (healthtechbridge.net), to facilitate direct engagement with stakeholders [15].

## Conclusions

This study validates the Biomarker Toolkit using an independent dataset and explores a different biomarker type (diagnostic versus prognostic), thereby contributing to the evidence base for its broader applicability. The Toolkit demonstrated its ability to distinguish between successful and stalled diagnostic colorectal cancer biomarkers, particularly in attributes related to clinical utility, even before regulatory approval. These findings support its potential value in predicting the likelihood of translational success. We aim for the continued application and evaluation of the Toolkit to contribute to more structured, transparent, and evidence-based approaches to biomarker development and clinical translation, whilst also helping to reduce the associated time and costs.

## Supplementary Information

Below is the link to the electronic supplementary material.

Supplementary Material 1

## Data Availability

All data generated or analysed during this study are included in this published article [and its supplementary information files]. A full data dictionary, with de-identified study-level scores, and the statistical R scripts will be made available via Zenodo.

## Abbreviations

ALU: ALU Based Cell Free DNA
AV: Analytical Validity
BEST: Biomarkers, EndpointS, and other Tools
CE: Cost-effectiveness
CRC: Colorectal Cancer
CU: Clinical Utility
CV: Clinical Validity
DA: Decisional Analysis
FDA: Food and Drug Administration
FI: Feasibility and Implementation
FIT: Fecal Immunochemical Test
Fn: Fusibacterium Nucleatum
gFOBT: Guaiac Fecal Occult Blood Test
HF: Human Factors
mSEPT9: methylated Septin9
NICE: National Institute for Health and Care Excellence
M2PK: M2 Pyruvate Kinase (M2PK)
Mir200c: MicroRNA-200c
Mir21: MicroRNA-21
