# Supplementary material for: Validation of the biomarker toolkit using diagnostic colorectal cancer biomarkers – an evidence-based tool to support clinical adoption of biomarkers
Source: J Transl Med. 2026 May 19;24:895. doi: 10.1186/s12967-026-08143-9 (PMC13366890; doi:10.1186/s12967-026-08143-9)
Supplement: Supplementary file 1 — Supplementary Material 1 [file 12967_2026_8143_MOESM1_ESM.docx]

**Additional file 1: Table S1** – Diagnostic Colorectal Cancer Biomarkers: MeSH Search Terms, Systematic Search Results, Abstracts and Full Papers Screened, and Classification of Papers

The table presents a modified PRISMA flow diagram, tabulated to provide a clearer and more effective representation of systematic searches for four successful and seven stalled diagnostic colorectal cancer biomarkers. A systematic search was conducted for each diagnostic CRC biomarker using biomarker specific MeSH terms (Additional file 1: Table S1) combined with colorectal cancer related terms (Additional file 1: Table S2) using the AND operator. For example, studies on the Guaiac Fecal Occult Blood Test were identified by searching for both the biomarker (Additional file 1: Table S1) AND colorectal cancer (Additional file 1: Table S2).

| **Diagnostic Colorectal Cancer Biomarkers** | **Search Terms** | **FDA approval year** | **Identified Articles** | **Abstracts Screened** | **Full articles assessed for eligibility** | **Included** | | |
| --- | --- | --- | --- | --- | --- | --- | --- | --- |
|  |  |  |  |  |  | **Selected articles** | **Extra articles** | **Types of articles selected** |
| Guaiac Fecal Occult Blood Test (gFOBT) | f?ecal occult blood or FOBT or f?ecal occult blood test or *Occult Blood or guaiac f?ecal occult or ?FOBT or f?ecal hb or f?ecal h?emoglobin or f?ecal blood | 1993 | 15815 | 10458 | 680 | 498 | 2 | 123 CS, 2 AV,  59 CE, 83 CU,  135 FI, 94 HF, 2 DA |
| Fecal Immunochemical Test  (FIT) | f?ecal immunochemical test* or FIT or *Occult Blood or f?ecal hb or f?ecal h?emoglobin or f?ecal blood or f?ecal immun* test* or iFOBT or immun* f?ecal occult blood test* | 1997 | 11834 | 7751 | 2009 | 929 |  | 509 CS, 47 AV,  94 CE, 58 CU,  193 FI, 50 HF, 23 DA |
| Cologuard | Cologuard* or multitarget stool DNA* or multi target stool DNA* or multi-target stool DNA* or multi?target stool DNA* or mtsDNA* or mt sDNA* or mt-sDNA* or mt?sDNA* or stool DNA* or sDNA* or s?DNA* or multitarget DNA* or multi-target DNA* or multi?target DNA* or NDRG4* or NDRG?4* or NDRG family member 4* or BMP3* or BMP?3* or bone morphogenetic protein 3* or KRAS* or K?RAS* or Kirsten rat sarcoma virus | 2014 | 1224 | 827 | 113 |  |  | 26 CS, 21 CU,  18 CE, 19 FI, 7 HF |
| Methylated Septin9 (mSEPT9) | SEPT9 or Septin-9 or SEPTIN9 or ?SEPT9 or methylated SEPT9 | 2016 | 1233 | 832 | 84 | 56 |  | 41 CS, 6 AV, 4 CE, 3 CU, 1 FI, 2 HF |
| ALU Based Cell Free DNA  (ALU) | Exp ALU elements or ALU* or (ALU* adj5 cfDNA) or (((ALU* adj5 cf DNA) or ALU*) adj5 cf?DNA) or (ALU* adj5 cell-free DNA) or (((ALU* adj5 cell?free DNA) or ALU*) adj5 cell?freeDNA) or (ALU* adj5 gene*) or ALU adj5 DNA or ALU adj5 repetitive ALU adj5 element or ALU adj5 element* or ALU adj5 family or ALU adj5 sequence or ALU adj5 nuclear or ALU adj5 retroelement or ALU adj5 retrotransposon or ALU adj5 transposon |  | 1431 | 631 | 39 | 22 |  | 18 CS, 4 AV |
| Anti-p53 Autoantibody (Anti-p53) | p53 antibodies / p53 antibody /  anti-p53 anti* / S-p53Ab / anti-p53 / anti-p53 antibody |  | 353 | 214 | 57 | 42 |  | 28 CS, 9 AV, 1 CU |
| Fusibacterium Nucleatum  (Fn) | fusobacterium nucleatum.mp. or Fusobacterium nucleatum/ or F* nucleatum.mp. |  | 1767 | 1156 | 65 | 53 |  | 43 CS, 4 AV, 6 CU, 1 FI, |
| M2 Pyruvate Kinase  (M2PK) | exp Pyruvate Kinase/ or (M2PK or M2-PK or M2 PK).mp. or M2?PK or (M2 adj5 pyruvate kinase) or (tM2-PK or tM2 PK or tM2PK) |  | 630 | 473 | 78 | 43 |  | 38 CS, 1 AV |
| MicroRNA-200c (Mir200c) | (mir-200c* or mir?200c*) or microRNA 200c* microRNA200c* or micro-RNA 200c* or micro?RNA?200c* or mir200c* or mir 200c* or mir-200c* or (miRNA-200c* or miRNA 200c*) or (mi-RNA200c* or mi-RNA 200c* or mi-RNA-200c*) or HAS-Mir-200c* |  | 1038 | 354 | 69 | 24 |  | 17 CS, 2 AV |
| MicroRNA-21 (Mir21) | (miR21* or miR 21* or miR?21* or miR-21*) or (microRNA 21* or microRNA21* or microRNA-21* or microRNA?21*) or (micro-RNA 21* or micro-RNA21*) or (miRNA-21* or miRNA21* or miRNA?21*) or (mi-RNA 21* or mi-RNA21* or mi-RNA-21*) or hsa-mir-21* |  | 5100 | 1882 | 154 | 89 |  | 75 CS, 12 AV, 3 CU |
| Transferrin | Transferrin / transferrin saturation / hTf / TF |  | 1071 | 783 | 28 | 13 |  | 11CS, 2 AV |

CS, clinical study; AV, analytical validity; CE, cost-effectiveness; CU, clinical utility; FI, feasibility and implementation; HF, human factors; DA, decisional analysis

In some cases, a study addressed multiple study types, thus categorised under each relevant category. Hence, discrepancy overall between total number of articles selected and number of each article type.

**Additional file 1: Table S2** – MeSH Search Terms for Colorectal Cancer

|  | **Medline** | |  | **Embase** | |
| --- | --- | --- | --- | --- | --- |
| 1 | exp Colorectal Neoplasms/ | 232817 | 1 | exp colon cancer/ | 124376 |
| 2 | exp Rectal Neoplasms/ | 53134 | 2 | exp rectum cancer/ | 57062 |
| 3 | (colorect* cancer* or colo-rect* cancer* or colo?rect* cancer* or colo* cancer* or rect* cancer*).mp. | 199044 | 3 | exp colorectal cancer/ | 374518 |
| 4 | (colorect* malignan* or colo-rect* malignan* or colo?rect* malignan* or colo* malignan* or rect* malignan*).mp. | 1619 | 4 | exp colon tumor/ | 170588 |
| 5 | (colorect* carcinoma* or colo-rect* carcinoma* or colo?rect* carcinoma* or colo* carcinoma* or rect* carcinoma*).mp. | 35269 | 5 | exp rectum tumor/ | 78905 |
| 6 | (colorect* adenocarcinoma* or colo-rect* adenocarcinoma* or colo?rect* adenocarcinoma* or colo* adenocarcinoma* or rect* adenocarcinoma*).mp. | 15463 | 6 | exp colorectal tumor/ | 450643 |
| 7 | (colorect* neoplas* or colo-rect* neoplas* or colo?rect* neoplas* or colo* neoplas* or rect* neoplas*).mp. | 222623 | 7 | exp colon carcinoma/ | 39212 |
| 8 | (colorect* tumo?r* or colo-rect* tumo?r* or colo?rect* tumo?r* or colo* tumo?r* or rect* tumo?r*).mp. | 17996 | 8 | exp rectum carcinoma/ | 16908 |
| 9 | (colorect* carcinogenesis or colo-rect* carcinogenesis or colo?rect* carcinogenesis or colo* carcinogenesis or rect* carcinogenesis).mp. | 6150 | 9 | exp colorectal carcinoma/ | 78781 |
| 10 | (colorect* oncogenesis or colo-rect* oncogenesis or colo?rect* oncogenesis or colo* oncogenesis or rect* oncogenesis).mp. | 51 | 10 | exp colon adenocarcinoma/ | 14208 |
| 11 | (colorect* tumorigenesis or colo-rect* tumorigenesis or colo?rect* tumorigenesis or colo* tumorigenesis or rect* tumorigenesis).mp. | 2007 | 11 | exp colorectal adenocarcinoma/ | 15305 |
| 12 | 1 or 2 or 3 or 4 or 5 or 6 or 7 or 8 or 9 or 10 or 11 | 207818 | 12 | exp colorectal neoplasms/ | 450643 |
|  |  |  | 13 | exp rectal neoplasms/ | 78905 |
|  |  |  | 14 | 9 or 10 or 11 or 12 or 13 or 14 or 15 or 16 or 17 or 18 or 19 or 20 or 21 | 450643 |
|  |  |  | 15 | colo* cancer*.mp. | 340912 |
|  |  |  | 16 | rect* cancer*.mp. | 59454 |
|  |  |  | 17 | (colorect* cancer* or colo-rect* cancer* or colo?rect* cancer*).mp. | 252616 |
|  |  |  | 18 | 23 or 24 or 25 | 381354 |
|  |  |  | 19 | colo* tumo?r*.mp. | 67060 |
|  |  |  | 20 | rect* tumo?r*.mp. | 20472 |
|  |  |  | 21 | (colorect* tumo?r* or colo-rect* tumo?r* or colo?rect* tumo?r*).mp. | 34759 |
|  |  |  | 22 | 27 or 28 or 29 | 82294 |
|  |  |  | 23 | colo* carcinoma*.mp. | 68147 |
|  |  |  | 24 | rect* carcinoma*.mp. | 18867 |
|  |  |  | 25 | (colorect* carcinoma* or colo-rect* carcinoma* or colo?rect* carcinoma*).mp. | 38503 |
|  |  |  | 26 | 31 or 32 or 33 | 83300 |
|  |  |  | 27 | colo* adenocarcinoma*.mp. | 25891 |
|  |  |  | 28 | rect* adenocarcinoma*.mp. | 5249 |
|  |  |  | 29 | (colorect* adenocarcinoma* or colo-rect* adenocarcinoma* or colo?rect* adenocarcinoma*).mp. | 8200 |
|  |  |  | 30 | 35 or 36 or 37 | 30580 |
|  |  |  | 31 | colo* malignan*.mp. | 2426 |
|  |  |  | 32 | rect* malignan*.mp. | 407 |
|  |  |  | 33 | (colorect* malignan* or colo-rect* malignan* or colo?rect* malignan*).mp. | 1476 |
|  |  |  | 34 | 39 or 40 or 41 | 2807 |
|  |  |  | 35 | colo* neoplas*.mp. | 18910 |
|  |  |  | 36 | rect* neoplas*.mp. | 3787 |
|  |  |  | 37 | (colorect* neoplas* or colo-rect* neoplas* or colo?rect* neoplas*).mp. | 13952 |
|  |  |  | 38 | 43 or 44 or 45 | 22266 |
|  |  |  | 39 | (colo* carcinogenesis or colo* oncogenesis or colo* tumorigenesis).mp. | 14900 |
|  |  |  | 40 | (rect* carcinogenesis or rect* oncogenesis or rect* tumorigenesis).mp. | 41 |
|  |  |  | 41 | (colorect* carcinogenesis or colorect* oncogenesis or colorect* tumorigenesis or colo-rect* carcinogenesis or colo-rect* oncogenesis or colo-rect* tumorigenesis or colo?rect* carcinogenesis or colo?rect* oncogenesis or colo?rect* tumorigenesis).mp. | 5582 |
|  |  |  | 42 | 47 or 48 or 49 | 14932 |
|  |  |  | 43 | 22 or 26 or 30 or 34 or 38 or 42 or 46 or 50 | 495690 |

**Additional file 1: Table S3** – Description of Each Clinical Utility Study Sub-type

| **Study Type** | **Definition** |
| --- | --- |
| Clinical Study | Assesses performance of selected biomarker for CRC diagnosis. |
| Assay Validation Study | Assesses the technical aspects of assay technology used for selected biomarker. It does not involve clinical samples, and outcome being assessed is not CRC diagnosis. |
| Clinical Utility Study |  |
| - Cost-effectiveness | Assesses additional cost benefits and incremental clinical utility achieved by use of selected biomarker compared to costs of comparable current clinical tools for CRC diagnosis. |
| - Human factor | Assesses participants’ opinions regarding use of selected biomarker. This includes exploring participants’ preference for using biomarker compared to other methods of CRC diagnosis and usability of biomarker for clinical testing. |
| - Decisional analysis | Assesses applicability of selected biomarker to patient pathway, including its potential to influence clinicians' diagnostic approaches. |
| - Feasibility/ implementation | Assesses utilisation of selected biomarker in real world conditions. This includes studies examining the impact of demographic and epidemiological factors, participation rates and barriers to adoption. |
| - Utility | Encompasses any other clinical utility studies not covered by the above sub-types. |

**Additional file 1: Table S4** – Toolkit Scores between Successful and Stalled Diagnostic Colorectal Cancer Biomarkers

|  | **Successful Biomarkers** | | | **Stalled Biomarkers** | | | **Mann Whitney U test** | **Binary Logistic Regression** | | | **Cox-regression Model** | | | |
| --- | --- | --- | --- | --- | --- | --- | --- | --- | --- | --- | --- | --- | --- | --- |
|  | Average Score (%) | SEM | D'Agostino-Pearson test | Average Score (%) | SEM | D'Agostino-Pearson test | P-value | Sig. | 95% C.I. for EXP(B) | | Sig. | Exp(B) | 95% C.I.  for EXP(B) | |
| **Categories** |  |  |  |  |  |  |  |  | Lower | Upper |  |  | Lower | Upper |
| Total | 44.7 | 0.58 | <.001 | 25.1 | 0.56 | <.001 | <.001 | <.001 | 3.16 x 10^9^ | 2.72 x 10^13^ | <.001 | 1383 | 456 | 4200 |
| Analytical Validity (AV) | 32.2 | 0.71 | 0.036 | 37.2 | 0.68 | 0.020 | <.001 | 0.187 | 0.004 | 3 | 0.214 | 0.502 | 0.169 | 1.49 |
| Clinical Validity (CV) | 27.2 | 0.66 | <.001 | 17.9 | 0.62 | <.001 | <.001 | <.001 | 223 | 1.69 x 10^5^ | 0.027 | 4.11 | 1.17 | 14.4 |
| Amended Clinical Utility (CU) | 74.6 | 1.43 | <.001 | 20.2 | 1.27 | <.001 | <.001 | <.001 | 3.60 x 10^3^ | 2.25 x 10^5^ | <.001 | 35.1 | 18.4 | 66.9 |
|  |  |  |  |  |  |  |  |  |  |  |  |  |  |  |
| **Sub-categories** |  |  |  |  |  |  |  |  |  |  |  |  |  |  |
| 4: Assay Validation |  |  |  |  |  |  |  | .002 | 52.4 | 4.55 x 10^7^ |  |  |  |  |
| 4b: Assay Validation |  |  |  |  |  |  |  | .004 | 0.008 | 0.404 |  |  |  |  |
| 5: Authority Approval |  |  |  |  |  |  |  | <.001 | 606 | 5.61 x 10^5^ | <.001 | 7.56 | 4.04 | 14.1 |
| 6: Biospecimen matrix/type |  |  |  |  |  |  |  | 0.038 | 1.21 | 597 |  |  |  |  |
| 7: Biospecimen collection technique |  |  |  |  |  |  |  | <.001 | 14.4 | 1.70 x 10^4^ | 0.010 | 3.26 | 1.33 | 8.04 |
| 9: Biospecimen Quality |  |  |  |  |  |  |  | 0.003 | 0 | .163 | 0.049 | 0.527 | 0.230 | 1.21 |
| 12: Decisional Analysis |  |  |  |  |  |  |  | 0.046 | 1.03 | 23.5 |  |  |  |  |
| 13: Ethics |  |  |  |  |  |  |  | 0.002 | 0.005 | .315 | <.001 | 0.431 | 0.280 | 0.662 |
| 28: Prespecified hypothesis |  |  |  |  |  |  |  | 0.014 | 1.91 | 285 | 0.011 | 2.027 | 1.18 | 3.50 |
| 32: Sample size calculation |  |  |  |  |  |  |  | 0.001 | 12.8 | 2.89 x 10^4^ |  |  |  |  |
| 33: Sensitivity/ specificity |  |  |  |  |  |  |  | 0.003 | 2.65 | 132 |  |  |  |  |
| 34: Statistical modelling |  |  |  |  |  |  |  |  |  |  | <.001 | 0.034 | 0.007 | 0.166 |
| 35: Storage/Shipping/ Transport |  |  |  |  |  |  |  | 0.005 | 14.3 | 3.86 x 10^6^ |  |  |  |  |
| 37: Time between diagnosis and sampling |  |  |  |  |  |  |  | 0.002 | 0 | .081 |  |  |  |  |
| 38: Trial design description |  |  |  |  |  |  |  | <.001 | 0 | .109 | <.001 | 0.368 | 0.205 | 0.662 |
| 39: Utility |  |  |  |  |  |  |  | 0.003 | 9.11 | 3.24 x 10^4^ | 0.004 | 7.32 | 1.88 | 28.4 |
| 40: Blinding |  |  |  |  |  |  |  |  |  |  | 0.016 | 1.74 | 1.11 | 2.72 |
| 41: Vital state of biospecimen |  |  |  |  |  |  |  | .048 | 0.005 | .979 |  |  |  |  |


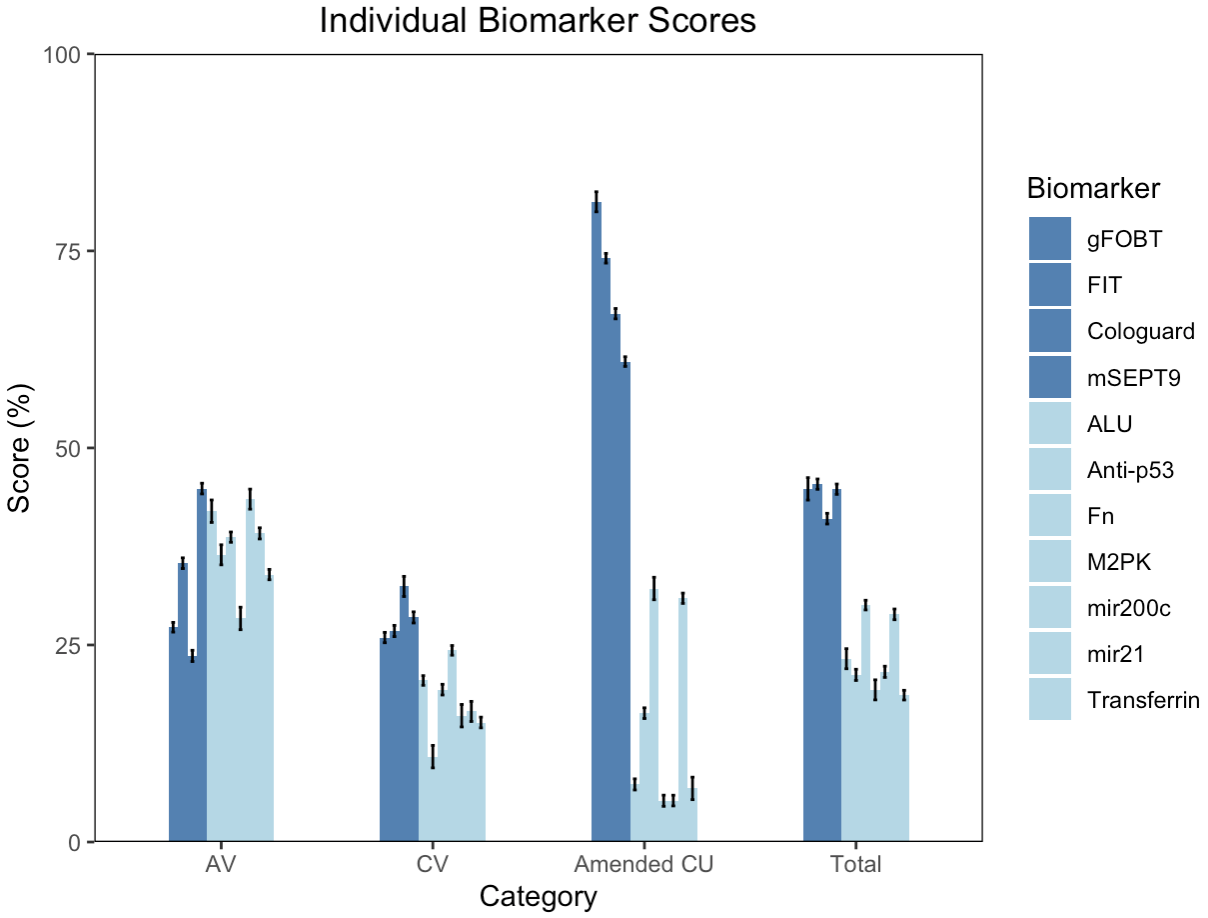


**Additional file 1: Figure S5** – Toolkit Scores for Each Biomarker

The first four bars represent the scores for successful diagnostic colorectal cancer biomarkers (gFOBT, FIT, Cologuard, mSEPT9) across four categories. The following seven bars represent the scores for stalled diagnostic colorectal cancer biomarkers (ALU, Anti-p53, Fn, M2PK, mir200c, mir21, Transferrin) across the same categories.

AV, Analytical Validity; CV, Clinical Validity; Amended CU, Amended Clinical Utility

**
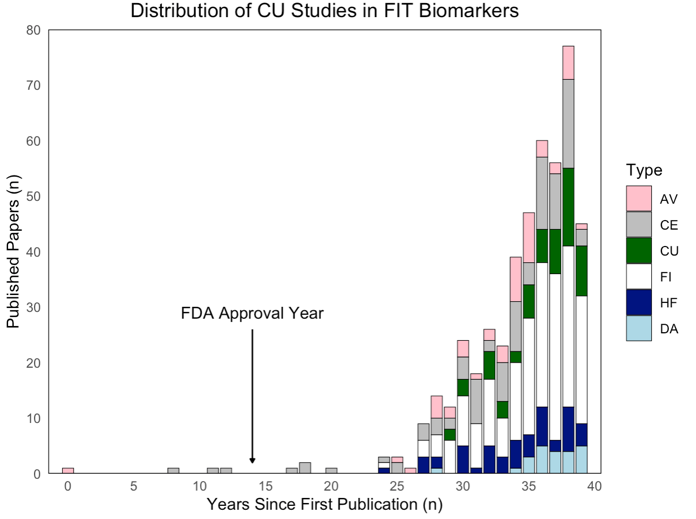

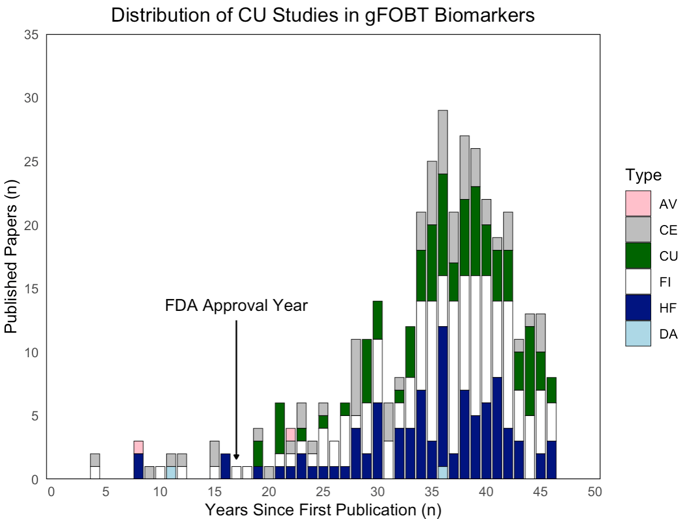

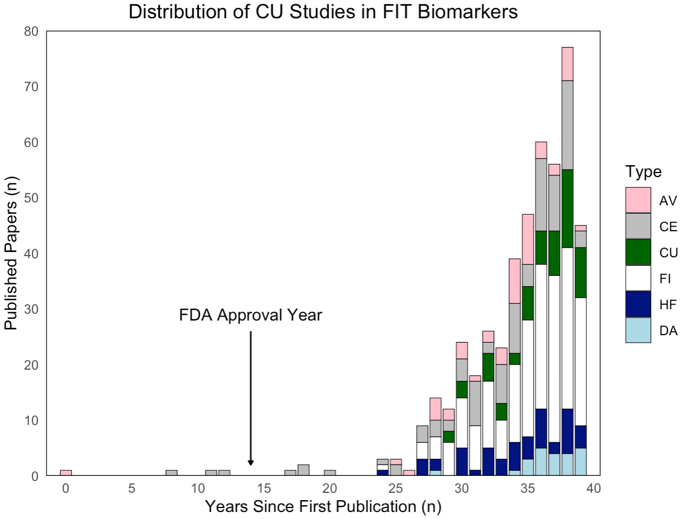
** **a**   **b**

**
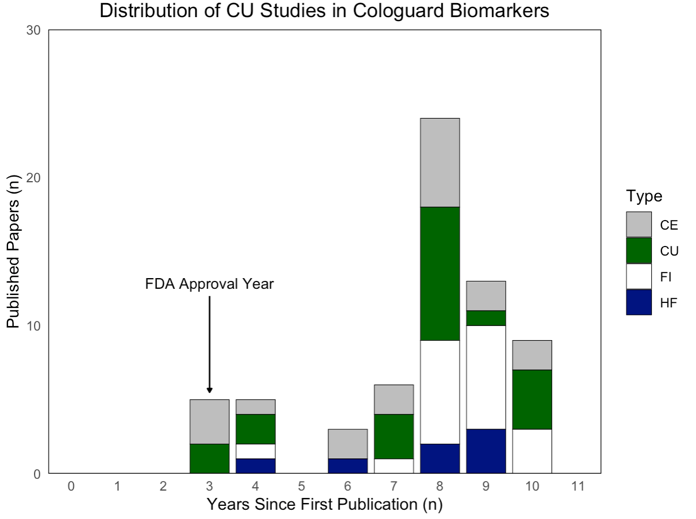
**
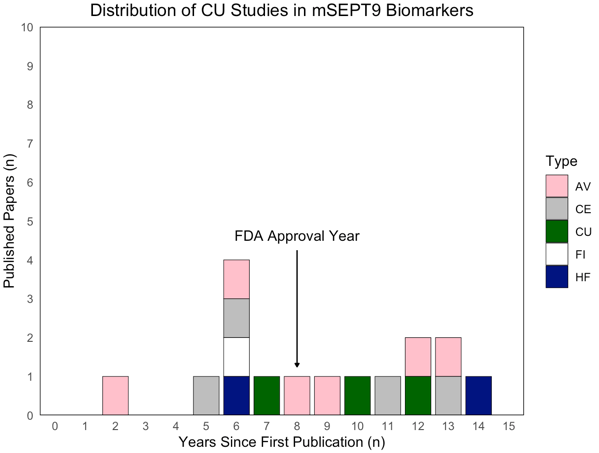


**
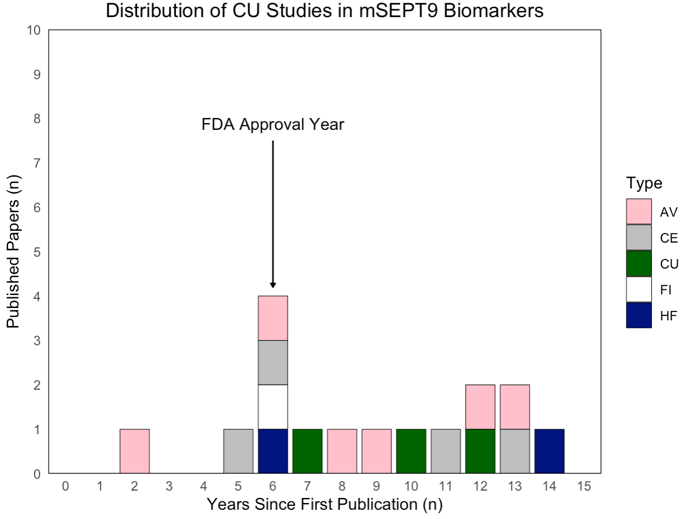
**
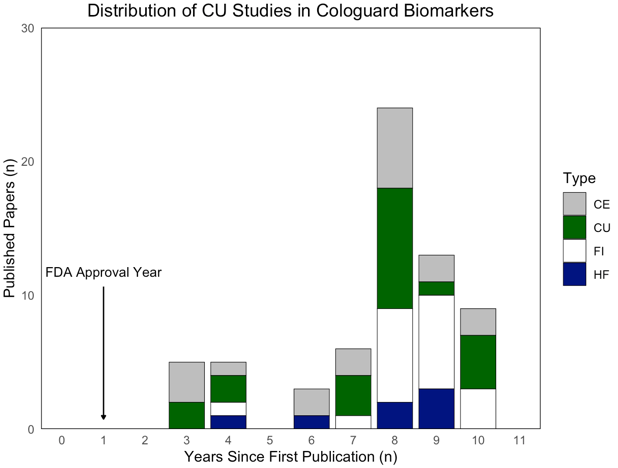
**c d**

**Additional file 1: Figure S6** – Publication Frequency of Clinical Utility Studies for Individual Successful Diagnostic Colorectal Cancer Biomarkers

The arrow indicates the number of years between the first publication of the relevant biomarker to its FDA approval.

**a** Publication frequency of gFOBT clinical utility studies

**b** Publication frequency of FIT clinical utility studies

**c** Publication frequency of mSEPT9 clinical utility studies

**d** Publication frequency of Cologuard clinical utility studies

AV, Analytical Validity; CE, Cost-effectiveness; CU, Clinical Utility; FI, Feasibility and Implementation; HF, Human Factors; DA, Decisional Analysis
